# Association between statin use, the vaginal microbiome, and *Gardnerella vaginalis* vaginolysin-mediated cytotoxicity

**DOI:** 10.1371/journal.pone.0183765

**Published:** 2017-08-28

**Authors:** Abdallah A. Abdelmaksoud, Philippe H. Girerd, Erin M. Garcia, J. Paul Brooks, Lauren M. Leftwich, Nihar U. Sheth, Steven P. Bradley, Myrna G. Serrano, Jennifer M. Fettweis, Bernice Huang, Jerome F. Strauss, Gregory A. Buck, Kimberly K. Jefferson

**Affiliations:** 1 Department of Microbiology and Immunology, Virginia Commonwealth University, Richmond, Virginia, United States of America; 2 Department of Obstetrics and Gynecology, Virginia Commonwealth University, Richmond, Virginia, United States of America; 3 Center for the Study of Biological Complexity, Virginia Commonwealth University, Richmond, Virginia, United States of America; Fred Hutchinson Cancer Research Center, UNITED STATES

## Abstract

**Background:**

Bacterial vaginosis (BV) is the leading dysbiosis of the vaginal microbiome. The pathways leading towards the development of BV are not well understood. *Gardnerella vaginalis* is frequently associated with BV. *G*. *vaginalis* produces the cholesterol-dependent cytolysin (CDC), vaginolysin, which can lyse a variety of human cells and is thought to play a role in pathogenesis. Because membrane cholesterol is required for vaginolysin to function, and because HMG-CoA reductase inhibitors (statins) affect not only serum levels of cholesterol but membrane levels as well, we hypothesized that statins might affect the vaginal microbiome.

**Methods:**

To investigate the relationship between use of the statins and the vaginal microbiome, we analyzed 16S rRNA gene taxonomic surveys performed on vaginal samples from 133 women who participated in the Vaginal Human Microbiome Project and who were taking statins at the time of sampling, 152 women who reported high cholesterol levels but were not taking statins, and 316 women who did not report high cholesterol. To examine the effect of statins on the cytolytic effect of vaginolysin, the cholesterol-dependent cytolysin (CDC) produced by *Gardnerella vaginalis*, we assessed the effect of simvastatin pretreatment of VK2E6/E7 vaginal epithelial cells on vaginolysin-mediated cytotoxicity.

**Results:**

The mean proportion of *G*. *vaginalis* among women taking statins was significantly lower relative to women not using statins. Women using statins had higher mean proportions of *Lactobacillus crispatus* relative to women with normal cholesterol levels, and higher levels of *Lactobacillus jensenii* relative to women with high cholesterol but not taking statins. In vitro, vaginal epithelial cells pretreated with simvastatin were relatively resistant to vaginolysin and this effect was inhibited by cholesterol.

**Conclusions:**

In this cross-sectional study, statin use was associated with reduced proportions of *G*. *vaginalis* and greater proportions of beneficial lactobacilli within the vaginal microbiome. The negative association between statin use and *G*. *vaginalis* may be related to inhibition of vaginolysin function.

## Introduction

The healthy vaginal microbiome is predominated by lactobacilli, which produce lactic acid and other toxic products and are associated with reduced bacterial growth and limit bacterial diversity and bioburden[1]. When *Lactobacillus* numbers are low, the vaginal pH is typically higher and the vaginal microbiome can become dominated by other bacterial taxa that are associated with bacterial vaginosis (BV). The magnitude of the negative correlation between lactobacilli and BV-associated bacteria is not equal among *Lactobacillus* species. There is a greater negative association between hydrogen-peroxide producing species of lactobacilli and BV[2], and, while it has been suggested that this association is not causal[3], H_2_O_2_-producing species such as *Lactobacillus crispatus*, and *Lactobacillus jensenii*, are less frequently observed than species that often lack the capacity to produce H_2_O_2_, including *Lactobacillus gasseri* and *Lactobacillus iners*, prior to BV onset[4,5]. Five common community state types, or CSTs have been described, four of which are predominated by lactobacilli: CST I (*L*. *crispatus*), II (*L*. *gasseri*), III (*L*. *iners*), and V (*L*. *jensenii*), and one by non-*Lactobacillus* taxa (CST IV)[6]. CST IV is characterized by higher pH, greater microbial diversity, and elevated Nugent score. Nugent’s scoring system is a test for BV in which a vaginal smear is gram-stained and bacterial morphotypes counts are used to calculate a score[7]. As an elevated Nugent score (7 or higher) is considered indicative of BV, CST IV is also associated with a positive BV diagnosis. Symptoms of BV may be mild or not reported, but this dysbiosis is associated with an increased risk for preterm birth and the acquisition of sexually transmitted infections, including HIV [8–13]. Women of African ancestry (AA) are more likely to exhibit CST IV whereas women of European ancestry (EA) are more likely to exhibit CST I [6], and they are more than twice as likely to have BV relative to EA [14]. This is important because AA have a 2–3 fold higher risk of giving birth very preterm (<32 weeks completed gestation) relative to EA, and are 20-times more likely to acquire HIV[15,16]. These substantial health disparities could be explained, at least partially, by differences in the vaginal microbiome [17]. One species that is significantly more abundant in the vaginal microbiome of AA women is *Gardnerella vaginalis* [14]. *G*. *vaginalis* is a common member of CST IV and its presence is associated with increased vaginal bacterial diversity[6].

*G*. *vaginalis* was first identified as the causative agent of BV but doubt was cast on this notion when it was discovered that healthy women are often colonized by *G*. *vaginalis* and vaginal challenge of women with pure cultures of the organism do not reliably cause BV [18,19]. Whether it is the causative agent or not, *G*. *vaginalis* has a very strong association with BV. It is present in nearly all cases of BV, and it forms biofilms on the vaginal epithelium that contribute to the poor cure rates of antimicrobial therapy[20–22]. In rare cases, it can be isolated as the sole infecting agent in bacteremia and osteomyelitis, and has a number of virulence properties, reaffirming its potential as an occasional opportunistic pathogen[23–27]. One of these virulence factors is the cholesterol-dependent cytolysin (CDC) vaginolysin (VLY)[28]. As with all CDC family members, VLY associates with cholesterol in the plasma membrane of host cells and forms large oligomeric pores and its activity is consequently dependent upon the presence of cholesterol in the membrane [29]. The role of cholesterol in CDC function is not fully understood, but studies have shown that high levels of cholesterol (50 mol%) are required for pore formation in lipid micelles and that CDCs fail to form pores at 40 mol% cholesterol [30].

Cholesterol trafficking in the human body is complex. It is synthesized by cells but can also come from dietary sources. It is in constant flux between intracellular compartments, the plasma membrane and extracellular compartments[31]. Low density lipoproteins (LDL) are used to shuttle cholesterol through the blood to cells and tissues in the body when it is required and high density lipoproteins are used to rid the body of cholesterol when it is in excess. High LDL levels are associated with cardiovascular disease. Hence, when high LDL levels are detected in serum, efforts are made to reduce them. Statins are pharmacologic agents that inhibit HMG-CoA reductase, an enzyme required for mevalonate synthesis. Interruption of the mevalonate pathway prevents the synthesis of downstream compounds including cholesterol. Consequently, statins reduce cholesterol synthesis and lower serum cholesterol, which reduces the risk for cardiovascular disease. While statins are designed to reduce extracellular cholesterol in blood in the form of low density lipoprotein, they can also reduce cholesterol in the plasma membranes of cells and erythrocyte membrane cholesterol levels are reported to decrease following initiation of statin therapy[32,33]. Some studies indicate an association between statin use and reduced severity of certain infections, including pneumonia, although there is also conflicting data[34,35]. A recent study found that, in vitro, simvastatin treatment of human airway epithelial cells reduced the pore-forming activity of penumolysin, a CDC produced by *Streptococcus pneumoniae* [36]. Because of previously reported effects of statins on certain infections, we hypothesized that their use might affect the vaginal microbiome. Because CDCs like VLY depend upon a high mol% of cholesterol in cellular membranes for their pore-forming activity, we hypothesized that statin use could interfere with VLY function, resulting in a vaginal ecology supporting healthy lactobacillus populations and reducing *G*. *vaginalis* abundance. We therefore investigated the relationship between statin use and the vaginal microbiome, and the effect of simvastatin on VLY activity.

## Materials and methods

### Participant recruitment

Subjects for this study were selected from the 4,306 women enrolled in the Vaginal Human Microbiome Project at VCU (VaHMP). Participants recruited from outpatient clinics at the Virginia Commonwealth University Medical Center and the Virginia Department of Health following written, informed consent from 2009–2013. The Institutional Review Boards for Human Subjects Research at VCU (Panel B) and the Virginia Department of Health reviewed and approved this study. Participants filled out a detailed questionnaire that included questions about ethnicity, education, employment, health habits, dietary habits, and sexual history. Clinicians also filled out a diagnosis form at the time of each visit that included information about the purpose of each visit, and any diagnoses. Inclusion criteria for VaHMP included women age at least 18 years of age who were able to provide informed consent and who were willing or already scheduled to undergo a vaginal examination using a speculum. The inclusion criterion for the subset of women included in this study was current statin use. Control groups included women who reported normal cholesterol levels and no statin use and women who reported high cholesterol levels and no statin use selected randomly from the VaHMP database. The control groups were matched for age and ethnicity to have the same proportionality for every 5 years amongst AA and EA groups. Statin use and non-use and cholesterol levels were initially ascertained by self-report and confirmed through medical record abstraction.

### Sampling and sample processing

Clinicians used CultureSwab EZ polyurethane foam swabs (BD) to obtain specimens from the mid-vaginal wall during a speculum examination. DNA was extracted from the swabs within 4 h of collection using a Powersoil kit (MoBio). Surveys of the 16S rRNA genes present in the samples were generated as part of the Vaginal Human Microbiome Project [37]. Sequences were classified using a local installation of RDP Classifier [38](0.8 cut-off) and the STIRRUPS, an analysis platform that employs the USEARCH algorithm combined with a curated vaginal 16S rRNA gene database to achieve species-level identification [39].

### 16S rRNA gene survey

The V1-V3 hypervariable regions of the bacterial 16S rRNA gene were amplified by PCR using barcoded primers. The 16S primers contain the A or B Titanium sequencing adapter (shown in italics), followed immediately by a unique variable (6–9 base) barcode sequence and finally the 5’ end of primer. The forward primer was a mixture (4:1) of the primers Fwd-P1 (5’—*CCATCTCATCCCTGCGTGTCTCCGACTCAG* BBBBBB AGAGTTYGATYMTGGCTYAG) and Fwd-P2 (5’—*CCATCTCATCCCTGCGTGTCTCCGACTCAG* BBBBBB AGARTTTGATCYTGGTTCAG). The reverse primer was Rev1B (5’–*CCTATCCCCTGTGTGCCTTGGCAGTCTCAG* ATTACCGCGGCTGCTGG). PCR products were sequenced using the Roche 454 GS FLX Titanium platform. These data were generated as part of the Vaginal Human Microbiome Project[37]. Raw sequence data from the project is available from the Short Read Archive at NCBI (projectID phs000256)[37]. We used a deep sequencing approach with a median 24,030 reads/sample. Samples with fewer than 5,000 reads were excluded from the analysis.

Reads that met the following criteria were processed: 1) valid primer and multiplex identifier sequences were observed; 2) less than 10% of base calls had a quality score less than 10; 3) the average quality score was greater than Q20; and 4) the read length was between 200 and 540 bases. Sequences were classified using a local installation of the RDP classifier (0.8 cutoff) and using STIRRUPS, an analysis platform that employs the USEARCH algorithm combined with a curated vaginal 16S rRNA gene database[39,40].

### Statistical analysis of 16S survey data

Sequencing read counts were converted to proportions for all samples to determine the percent of the total microbiome that each bacterial species contributed. The predominant taxon in a sample refers to the taxon for which the largest number of reads were assigned taxonomic classification with confidence (i.e. the highest percentage of reads in the sample). Microbiomes were categorized by community state types (CST) similar to a previous study [6]. CST I, microbiomes in which the proportion *L*. *crispatus* > = 30% and predominant taxon = *L*. *crispatus*; CST II, proportion *L*. *gasseri* > = 30% and predominant taxon = *L*. *gasseri*; CST III, proportion *L*. *iners* > = 30% and predominant taxon = *L*. *iners*; CST IV, no proportions of any one species of *Lactobacillus* > = 30%; CST V, proportion *L*. *jensenii* > = 30% and predominant taxon = *L*. *jensenii*.

Linear discriminant analysis effect size (LEfSe) applies a Kruskal-Wallis rank sum test for each bacterium, then uses linear discriminant analysis to estimate effect size [41]. The effect size is the contribution of a variable to the ability to distinguish two different groups. The barplot indicating the effect size of bacterial species that correlate with statin use was generated through LEfSe using a minimum cut-off LDA score of three and reducing permutations to 100,000 to avoid very low abundance organisms.

The boxplot of *G*. *vaginalis* has whiskers that extend to the highest/lowest value within 1.5 times the interquartile range. Data beyond the end of the whiskers are outliers and are plotted as points. A Wilcoxon rank sum test with continuity correction was used to test whether the median and mean proportions of *G*. *vaginalis*, *L*. *crispatus*, and *L*. *jensenii* followed the same distribution for groups of subjects (Statin/no statin use high cholesterol/ no statin use low cholesterol, African/European ancestry). Analysis was conducted and plots were created using the R language for statistical computing and packages ggplot2 [42].

### Media and culture conditions

*G*. *vaginalis* strain AMD was grown in brain heart infusion supplemented with 10% human serum anaerobically at 37°C. *E*. *coli* was grown at 37°C under atmospheric conditions. The human vaginal epithelial cell line VK2/E6E7[43] was obtained from ATCC (ATCC^®^-CRL-2616^™^) and cultured at 37°C and 5% CO_2_ in Keratinocyte-Serum Free medium with 0.1 ng/ml human recombinant EGF, 0.05 mg/ml bovine pituitary extract, and additional calcium chloride 44.1 mg/L (final concentration 0.4 mM).

### Expression and purification of recombinant VLY

DNA was extracted from *G*. *vaginalis* strain AMD using the DNeasy Blood and Tissue kit (Qiagen). The *vly* gene was amplified using primers VaginolysinFWD (5’-GGAAGGGATCCGATTCTTCTGCAAAGCCTTCTGC-3’) and VaginolysinREV (5’-GGAAGCTCGAGTCAGTCATTCTTTACAGTTTCAGCAAC-3’) as previously described [28]. Purified PCR product was restricted with BamHI and XhoI and ligated to pET32. Plasmid from a colony that grew on LB agar containing 100 μg ampicillin / mL was confirmed by DNA sequencing and transformed into *E*. *coli* strain BL21(DE3) CodonPlus pRIPL (Agilent technologies). Cultures were grown in 1 L LB containing 100 μg amp / mL and 35 μg chloramphenicol / mL to exponential phase, induced with 1 mM IPTG for 2 hours, and the bacteria were collected by centrifugation. Bacteria were lysed in a French pressure cell in B-PER^™^ (ThermoFisher Scientific) containing protease inhibitors (EDTA-free cOmplete^™^, Sigma) and the lysate was cleared by centrifugation and filtration. The protein was purified by cobalt affinity chromatography (His-Pur^™^, Thermo-Fisher Scientific) according to manufacturer instructions, eluted in 0.25 M imidazole, and dialyzed against 1X phosphate buffered saline (PBS). The affinity tag was removed by thrombin digestion (Sigma-Aldrich, Thrombin CleanCleave Kit).

### Cytotoxicity assay

VK2/E6E7 cell monolayers were established in 96-well plates in 100 μL Keratinocyte media (Life Technologies Keratinocyte-SFM) per well. Once the monolayers reached 70% confluence, a fresh stock solution of 1 mg simvastatin / ml ethanol was prepared and monolayers were pre-treated with a final concentration of 1 μg simvastatin / ml or an equal volume (1 μl / ml) of ethanol for 48 hours in the presence or absence of 5 μg cholesterol / ml (Sigma, cholesterol balanced with methyl-ß-cyclodextrin) or 1mM mevalonolactone (Sigma) dissolved in ddH_2_O. Following the 48 hr pretreatment, media was replaced with fresh media containing ethanol or simvastatin but no serum or any other source of cholesterol, and purified recombinant VLY at 10 μg / ml or 5 μg / ml was incubated with the monolayers for 60 min at 37°C, trypan blue staining was performed, and pictures were immediately taken using a digital camera mounted on a light microscope. Samples that were not treated with VLY were included to confirm that the pre-treatment had no measurable effect on cell viability. Media from unstained replicate wells was analyzed using the Cytotoxicity Detection Kit for quantification of extracellular lactate dehydrogenase (Roche). The experiments were performed 3 times and each experimental replicate contained technical triplicate samples. Results for individual LDH assays were compared using one-way analysis of variance (ANOVA) with Tukey post-test for comparison of individual groups.

## Results

### Statin use is associated with vaginal community state type

**Fig 1:**
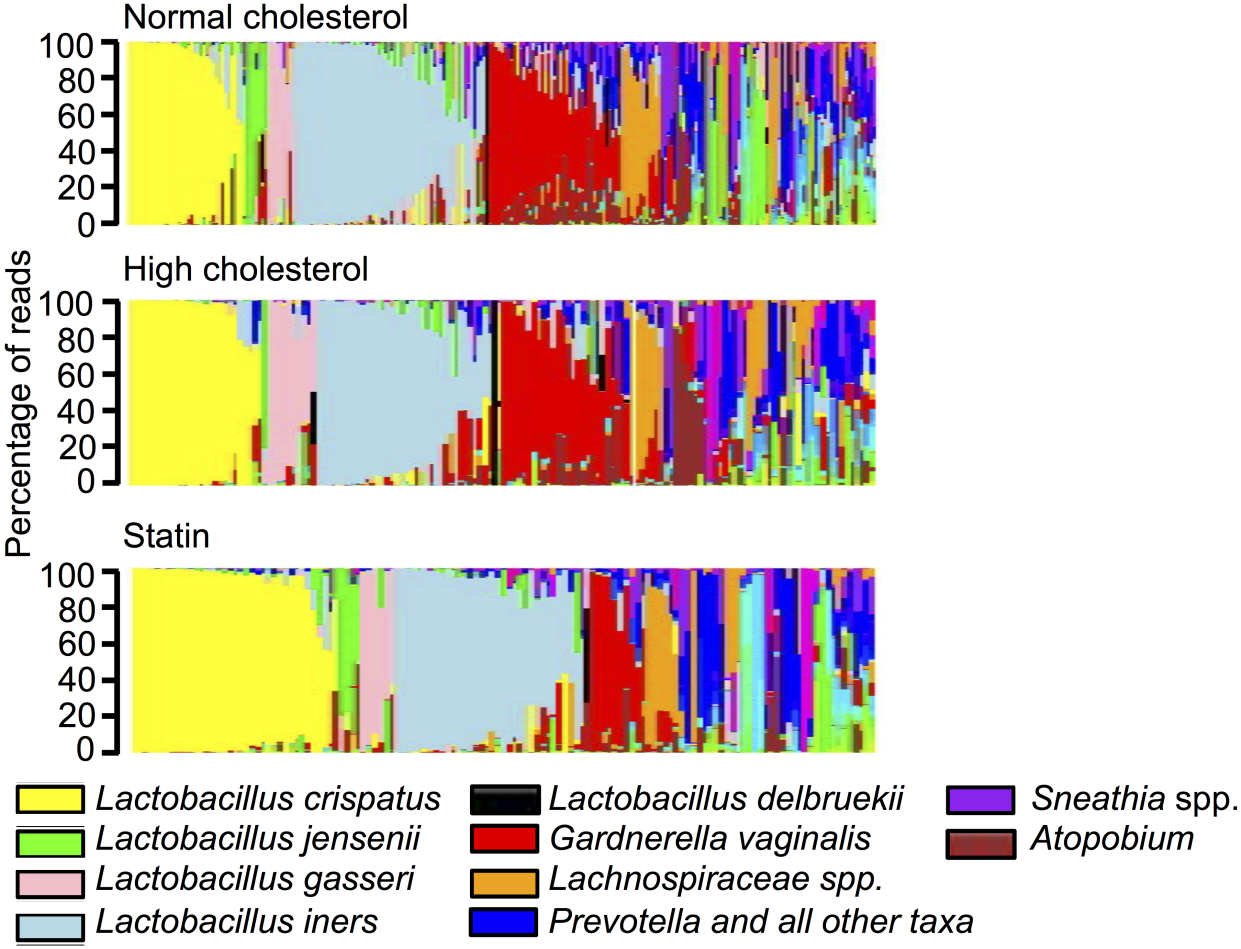
Microbial community profiles of women grouped by statin use. Stacked bar plots showing vaginal microbial community profiles from 316 women who did not report high cholesterol and who were not taking statins, 152 women reported having high cholesterol but who were taking statins, and 133 women who were taking statins. The profiles are grouped by the most abundant species and are ordered by decreasing proportion of the dominant bacterium.

We analyzed the 16S rRNA survey profiles of vaginal swabs from 72 AA and 61 EA women who were using statins at the time of sampling, 83 AA and 69 EA women who reported high cholesterol but were not taking statins, and 160 AA and 156 EA women who did not report high cholesterol and were not taking statins. Information about the subject groups is listed in Table 1. Tukey multiple comparison indicated that within the ethnic groups, there were no significant differences in any of the variables considered, including mean age, pregnancy status, douching, number of sexual partners, smoking, hormone replacement therapy, alcohol consumption, income, or other variables listed in Table 1, between statin users, women with high cholesterol but no statin use, and women who did not have high cholesterol. Fig 1 displays the vaginal microbiomes of the subjects, ordered first by predominant species comprising at least 30% of the sample and second, by proportion of the predominant species. The statin-using group appeared to have a higher overall proportion of the healthy *Lactobacillius* species *L*. *crispatus* and high diversity profiles were less common in this group relative to those who were not taking statins, regardless of cholesterol level (Fig 1). We used a system of categorizing community state types (CST) that was similar to what has been published previously [6] and found that CST I (*L*. *crispatus*) was significantly more common among AA who were taking statins users relative to those who were not taking statins, regardless of whether they reported normal (Fisher test p = 0.0042) or high cholesterol (p = 0.0297) (Table 2). Furthermore, CST IV was significantly less common among AA statin users versus non-statin users relative to non-statin users with both normal (p = 0.0068) and high cholesterol levels (p = 0.0099). There was a similar trend among EA statin users but the increase in CST I and the decrease in CST IV were not significant in this group. Because prior studies suggest that the vaginal microbiome changes following menopause, resulting in lower proportions of lactobacilli, we divided the cohorts into pre- and postmenopausal, based primarily on self-report (of the few women who did not report menopausal status, their menopausal status was predicted based on the average age of 51 years for menopause in the U.S.). The trend of higher rates of CST I and lower rates of CST IV among AA women taking statins remained, however, the only comparisons that were still significant were numbers of CST I among premenopausal AA women taking statins versus those with normal or high cholesterol but not taking statins (p = 0.0177 and p = 0.0217, respectively) and numbers of CST IV among premenopausal AA women taking statins versus those with normal cholesterol and not taking statins (p = 0.0305).

**Table 1:** Information about study participants.

	AA statin yes^1^	AA high cholesterol/ no statin^1^	AA normal cholesterol/ no statin^1^	EA statin yes^1^	EA high cholesterol/ no statin^1^	EA normal cholesterol/ no statin^1^
**Participants n**	72	83	160	61	69	156
**Age (mean)**	51.6	51.9	50.9	51.1	50.9	50.3
**Post-menopausal**^2^	47(65%)	51(61%)	103(65%)	34(56%)	35(51%)	77(49%)
**Menstrual cycle phase (days last period)**						
Menstruation (day 1–5)	2(3%)	3(4%)	5(3%)	3(5%)	3(4%)	4(3%)
Follicular phase (day 6–14)	4(6%)	3(4%)	7(4%)	3(5%)	5(7%)	9(6%)
Luteal phase (day 15–28)	6(8%)	6(7%)	10(6%)	5(8%)	5(7%)	13(8%)
>28 days	4(6%)	5(6%)	11(7%)	4(7%)	7(10%)	9(6%)
NA	57(79%)	66(80%)	127(80%)	46(75%)	49(71%)	121(77%)
**Taking hormonal replacement therapy**	4(6%)	3(4%)	14(9%)	5(8%)	5(7%)	16(10%)
**Pregnant**	2 (3%)	2 (2%)	1 (1%)	1 (2%)	1 (1%)	4 (3%)
**Douche in last month**	7(14%)	11 (19%)	24 (15%)	6 (10%)	4 (6%)	3 (2%)
**Sex partners past year**						
0	15 (21%)	19 (24%)	31 (19%)	13 (21%)	14 (20%)	26 (17%)
1	34 (47%)	42(53%)	80 (50%)	34 (59%)	34 (49%)	89 (57%)
>1	11 (15%)	7 (9%)	23 (14%)	7 (11%)	7 (10%)	8(5%)
N/A	12(17%)	15(18%)	26(16%)	7(11%)	14(20%)	33(21%)
**Current smoker**	27 (38%)	32 (40%)	66 (41%)	16 (27%)	24 (35%)	13 (36%)
**Yogurt consumption >1 per week**	26 (36%)	39 (49%)	65 (41%)	33 (55%)	37 (54%)	92 (72%)
**Alcohol >0 past week**	24 (33%)	20 (25%)	48 (30%)	22 (37%)	22 (32%)	67 (49%)
**Income**						
<15K	32 (44%)	40 (48%)	57 (36%)	14 (23%)	15 (22%)	14 (10%)
15K-20K	7 (10%)	11 (13%)	21 (13%)	2 (3%)	6 (9%)	3 (2%)
20K-40K	14 (19%)	13 (16%)	41 (26%)	5 (8%)	7 (10%)	17 (12%)
40K -60K	4 (6%)	7 (8%)	5 (3%)	8 (13%)	7 (10%)	26 (19%)
60K-80K	2 (3%)	2 (2%)	6 (4%)	12 (20%)	12 (17%)	17 (12%)
>80K	4 (6%)	3 (4%)	10 (6%)	17 (28%)	20 (29%1)	59 (43%)

Parameters listed in the table were self-reported.

^**1**^Statin use or non-use was confirmed by medical record abstraction.

^**2**^A small number of women did not report menopausal status and among this group, menopause was predicted based on age (51 years).

**Table 2:** Statin use affects community state type.

Menopausal status	Community state type	AA statins	AA high cholesterol	AA normal cholesterol	EA statins	EA high cholesterol	EA normal cholesterol
**All**	**CST I**	28% (20)	13% (11)	12% (19)	26% (16)	19% (13)	19% (26)
	**CST II**	3% (2)	5% (4)	1% (1)	10% (6)	7% (5)	8% (11)
	**CST III**	29% (21)	23% (19)	29% (46)	21% (13)	22% (15)	23% (31)
	**CST IV**	37% (27)	59% (49)	58% (92)	38% (23)	51% (35)	46% (83)
	**CST V**	3% (2)	0	1% (2)	5% (3)	1% (1)	4% (5)
**Pre**	**CST I**	36% (9)	9% (3)	12% (7)	33% (9)	29% (10)	20% (16)
	**CST II**	4% (1)	3% (1)	2% (1)	4% (1)	6% (2)	6% (5)
	**CST III**	25% (6)	28% (9)	24% (15)	26% (7)	17% (6)	21% (17)
	**CST IV**	32% (8)	60% (19)	60% (34)	33% (9)	49% (16)	48% (38)
	**CST V**	0% (1)	0% (0)	0% (0)	4% (1)	0% (0)	4% (3)
**Post**	**CST I**	23% (11)	16% (8)	12% (12)	21% (7)	9% (3)	13% (10)
	**CST II**	2% (1)	6% (3)	0% (0)	15% (5)	9% (3)	8% (6)
	**CST III**	32% (15)	20% (10)	30% (31)	18% (6)	26% (9)	18% (14)
	**CST IV**	40% (19)	59% (30)	56% (58)	41% (14)	54% (19)	62% (45)
	**CST V**	2% (1)	0% (0)	2% (2)	6% (2)	3% (1)	3% (2)

### Statin use is associated with decreased abundance of *G*. *vaginalis*

**Fig 2:**
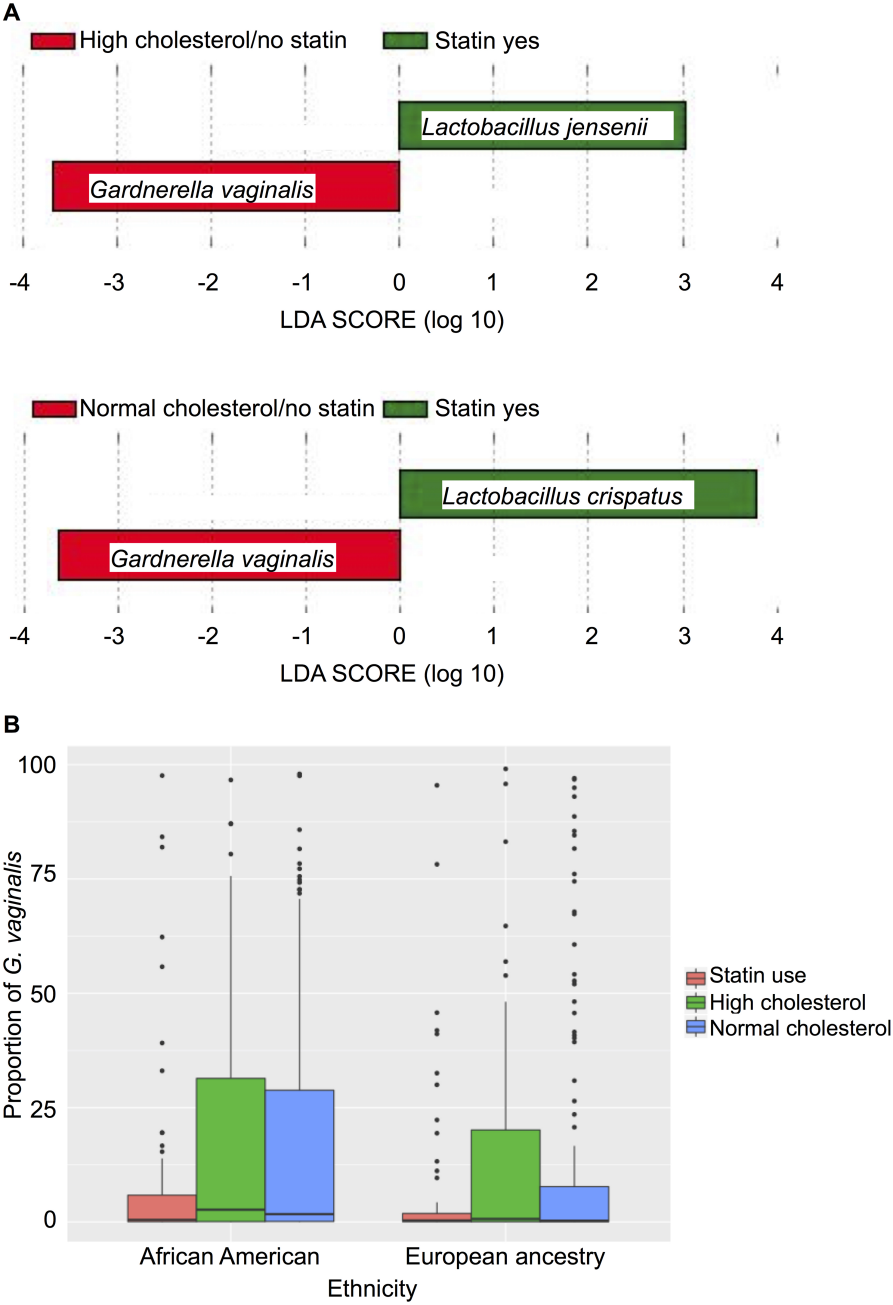
The proportion of *G*. *vaginalis* is lower in statin users. A. Taxa that occurred in significantly different proportions in the vaginal microbiomes of statin users were detected by LEfSE analysis. Taxa significantly higher in women taking statins are in green and the species significantly lower (*G*. *vaginalis*) is in red. The chart on top compares vaginal microbiota from women with high cholesterol who were not taking statins to women taking statins and the lower chart compares vaginal microbiota from women with normal cholesterol who were not taking statins to women taking statins B. Boxplot of *G*.*vaginalis* proportions in subjects grouped based on ethnicity and sub-grouped based on statin use and normal versus high cholesterol, with whiskers that extend to the highest/lowest value within 1.5 times the interquartile range, outliers beyond the whiskers are plotted as points. The horizontal line in each box indicates the median. A Wilcoxon rank sum test with continuity correction was used to test whether the proportion of BV-associated bacteria followed the same distribution for groups of subjects (statin/high cholesterol no statin/no high cholesterol, African/European ancestry).

We used linear discriminant analysis (LDA) effect size (LEfSe) to determine whether specific bacterial taxa were significantly associated with statin use. Fig 2A illustrates that, according to LEfSe, the proportion of *G*. *vaginalis* was significantly lower in statin users (8.3%) relative to non-statin users with either normal (15.6%; Wilcoxon p = 0.026) or high cholesterol (16.7%; p = 0.047). In contrast, the proportion of *L*. *jensenii* was greater in statin users (3.9%) relative to those who reported high cholesterol but were not taking statins (2.9%; p = 0.011) whereas the proportion of *L*. *crispatus* was higher in statin users (23.9%) relative to those who did not report high cholesterol (14.7%; p = 0.013) and were not taking statins. The mean proportion of *L*. *crispatus* in those who reported high cholesterol but were not taking statins was17.3%, which followed a similar trend but did not reach statistical significance (p = 0.068). Fig 2B shows the relationship between the proportion of *G*. *vaginalis* and statin use and ethnicity. And reveals a negative association between statin use and colonization by *G*. *vaginalis* relative to AA women with normal cholesterol levels who were not using statins (p = 0.0083) and AA women with high cholesterol who were not using statins (p = 0.0439).

### Simvastatin treatment protects vaginal epithelial cells from VLY

**Fig 3:**
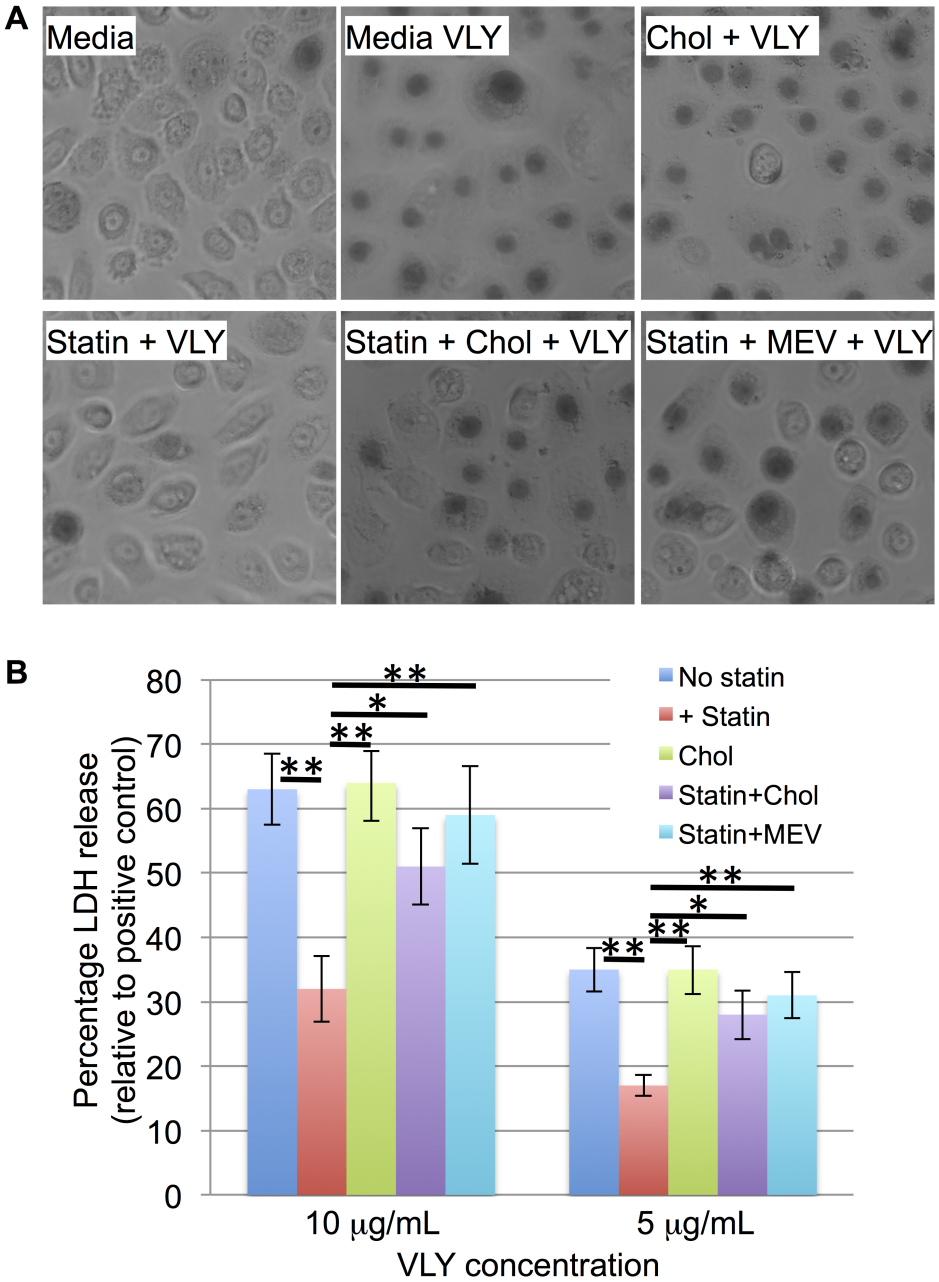
Statins reduce vaginolysin-mediated cytotoxicity. A) VK2/E6E7 vaginal keratinocytes were incubated in control media or media containing 5 μg cholesterol / ml (Chol), 1 μg simvastatin / ml (Statin), simvastatin and cholesterol, or simvastatin and 1mM mevalonate for 48 hours. The cells were then left unchallenged or challenged with 10 μg VLY / mL for 1 hour. Trypan blue staining was performed to monitor rounding and permeabilization (observed as central darkening of the cells). B) VK2/E6E7 cells pre-treated with control, simvastatin, cholesterol, simvastatin and cholesterol, or simvastatin and mevalonate, and then challenged with 10 or 5 μg VLY / mL. Lactate dehydrogenase release assay was used to quantify permeabilization of the cells. * p<0.05, ** p<0.005 using one-way ANOVA with Tukey post-test for comparison of individual groups.

We hypothesized that the decrease in the proportion of *G*. *vaginalis* observed in the statin treatment group could be due, in part, to an affect of host plasma membrane cholesterol depletion on the function of the cholesterol-dependent cytolysin, vaginolysin (VLY). To test this, we treated VK2/E6E7 vaginal keratinocytes with simvastatin, challenged them with purified, recombinant VLY, and assessed loss of membrane integrity and viability by trypan blue staining and lactate dehydrogenase release assay. One-way ANOVA with Tukey post-test for comparison of individual groups indicated a significant decrease in VLY-induced LDH release from simvastatin pre-treated cells. Treatment of the cells with either cholesterol or mevalonate (exogenous sources for the cells to assimilate in the absence of biosynthesis) in addition to the simvastatin, reduced the effect, and there was no longer a significant difference relative to untreated cells (p>0.05). Both trypan blue staining and LDH assay indicated that simvastatin pretreatment reduced VLY-mediated toxicity and that this effect was reversed by the addition of cholesterol or mevalonate during the pre-treatment (Fig 3).

## Discussion

In this study we found that statin use was positively associated with CST I and negatively associated with CST IV. Because the risk of poor cardiovascular health, and therefore the likelihood that one will be taking statins, increases with age, slightly more than half of the study cohort was postmenopausal. There is evidence to support that decreased estrogen following menopause leads to a decrease in glycogen accumulation in vaginal epithelial cells, and that this, in turn, leads to lower numbers of vaginal lactobacilli[44]. In our study we found that, among women not taking statins, the number of EA women with vaginal CST I was significantly lower in the postmenopausal subset, in agreement with a prior study in which the majority of the cohort was Caucasian[45]. In contrast, there was not a significant difference in the proportion CST I or CST IV among premenopausal versus postmenopausal AA women (regardless of cholesterol level or statin use). A previous study, in which the cohort was predominantly of African ancestry, noted that, while the numbers of vaginal lactobacilli were lower among postmenopausal women, the difference was not significant[46]. Thus, there may be less of an affect of menopausal status among AA women. In addition, the pre- and post-menopausal sub-cohorts in the current study may have been closer in age than the groups in prior studies. The trend of increased prevalence of CST I and decreased prevalence of CST IV among women taking statins remained when pre- and post-menopausal subjects were divided, however, the difference was only significant among premenopausal AA women. This may be because the number of women in the postmenopausal group was too small to achieve sufficient statistical power.

In addition to the association between vaginal CST and statin use, we found that the mean proportion of *G*. *vaginalis* was lower in the vaginal microbiomes of women who used statins relative to those of women who were not using statins. The effect was similar regardless of whether the women not using statins reported high or normal serum levels of cholesterol. This retrospective analysis cannot establish cause and effect, and it is possible that other factors could be at play. For example, there may exist behavioral differences between women with high cholesterol who are not using statins and women who are using statins, such as likelihood of seeking healthcare. Nonetheless, in this study we sought a mechanism through which statins could plausibly affect the proportion of *G*. *vaginalis*. Statins affect not only serum cholesterol levels, but membrane cholesterol as well. From the data collected through this study, we were unable to assess membrane cholesterol levels or to determine whether or not there is a relationship between serum levels and membrane levels. Possibly as a consequence of the decreased *G*. *vaginalis*, or through a more direct affect of statin use, women taking statins had significantly greater mean proportions of *L*. *crispatus* and *L*. *jensenii* relative to non-statin users with normal cholesterol levels and non-statin users with elevated cholesterol levels, respectively.

In vitro analysis of membrane permeabilization, as measured by trypan blue staining and LDH release by VLY in vaginal epithelial cells in vitro indicated that simvastatin-treatment was protective, suggesting a mechanism for the reduction in prevalence of *G*. *vaginalis* in the vaginal environment. The protective effect was reversed by the addition of either cholesterol or mevalonate, suggesting, as expected, that the statin effect was related to reduced mevalonate and subsequent cholesterol synthesis. Two facts suggest that VLY function may be essential for colonization by *G*. *vaginalis*. First, VLY is highly conserved in *G*. *vaginalis*, despite considerable genetic diversity in this species [28,47]. Second, other CDC’s, such as pneumolysin and listeriolysin, play critical roles in colonization and pathogenesis [48,49]. *G*. *vaginalis* has been shown to compete with *L*. *crispatus* for adherence to vaginal epithelial cells [50], therefore, the increase in the proportion of lactobacilli in women taking statins may be more than a statistical consequence of the decrease in *G*. *vaginalis*. *G*. *vaginalis* could also alter the conditions within the vagina in other ways that reduce lactobacilli or compete for resources, including lactic acid. Vaginal epithelial cells store glycogen, and glycogen is strongly associated with colonization by beneficial lactobacilli such as *Lactobacillus crispatus*. VLY can damage vaginal epithelial cells, likely depleting glycogen stores, which would be expected to reduce growth of beneficial lactobacilli and create an environment conducive to the growth of *G*. *vaginalis* and other BV-associated bacteria. Therefore, interference with VLY function through depletion of plasma membrane cholesterol, could prevent *G*. *vaginalis* growth and promote the growth of lactobacilli.

A recent study by Statt et al. found that human airway epithelial cells, treated with simvastatin or pravastatin, were resistant to pore-formation by the CDC, pneumolysin (PLY)[36]. As a CDC, PLY binding and activity are dependent upon cholesterol in the plasma membrane of target cells. The study found that, while pore-forming activity was inhibited, binding of PLY to epithelial cells was not affected by statin treatment, even though statin treatment did reduce plasma membrane cholesterol[36]. Other studies have also shown that a high cholesterol mol% is required for pore formation, but that binding still occurs, but fails to lead to pore formation at a lower mol%[30]. In our study, the lack of an antibody against VLY precluded our analysis of VLY binding to the statin-treated cells. Furthermore, we did not directly assess pore-formation by VLY, although trypan blue staining and LDH release are indicators of a decrease in membrane integrity.

In addition to the reduction of cholesterol synthesis, statins mediate a variety of effects on cells. They have anti-inflammatory effects and have been shown to reduce the pathology and severity of a number of infectious and autoimmune diseases[51–53]. Therefore, if the association between statin use and vaginal CST is indeed causal, it is plausible that anti-inflammatory effects also play a role.

AA women are twice as likely to have BV relative to EA women and they are more likely to have a vaginal microbiome that falls into CST IV. EA women are more likely to be colonized by *L*. *crispatus* and have a microbiome that falls into CST I. This is clinically relevant because BV-like vaginal microbial profiles are associated with preterm birth, which is more common in AA women, and HIV acquisition[54,55]. Studies suggest that this disparity cannot be accounted for by differences in demographics, suggesting that genetics plays a role[56]. Interestingly, women using statins exhibited similar CST profiles regardless of ethnicity, suggesting that it could potentially reduce the ethnic disparity in rates of BV. Furthermore, a more mechanistic understanding of the basis for higher rates of BV among AA women could help to reduce this and associated health disparities. The basis for the greater association between statin use and the vaginal microbiome in AA women is not clear from this study. Statins are significantly less effective at reducing LDL in AA women, suggesting that there may be a difference in cholesterol metabolism or in the effect of statins in this group[57,58]. This suggests that there may be physiological differences in the way that cholesterol is metabolized or trafficked between the two ethnic groups. Clinically, when cholesterol levels are measured, they are measured in serum, not in cellular plasma membranes. Cholesterol levels that would be expected to affect VLY function are the levels in cell membranes, not in serum. This may explain why ethnic-based differences in cholesterol metabolism have not been previously noted.

Strengths of this study include the use of comprehensive 16S rRNA gene survey and the relatively large sample size; 133 women taking statins, 316 women in the normal cholesterol level control group, and 152 women in the high cholesterol level control group. Because of the variability of the vaginal microbiome among women, significant effects of environmental factors are not easily detectable in smaller sample sets. Another strength is the in vitro component, which revealed a potential mechanism for the basis of decreased *G*. *vaginalis* abundance in women using statins. A limitation of the study is the retrospective nature of the analysis. A prospective analysis of the 16S profiles before and after initiation of statin treatment would have better isolated the effects of the statins. Another limitation is the lack of an animal model, which would allow for standardized exposure levels. Existing animal models are problematic as most of the bacterial taxa that make up the human vaginal microbiome do not colonize animals readily and VLY is specific for human cells.
